# Mr. Marson, on Burns and Scalds

**Published:** 1807-02

**Authors:** W. Marson

**Affiliations:** Worksop, Notts


### *To the Editors of the Medical and Physical Journal.*

GENTLEMEN,

WHATEVER may be the mode of action of the Oleum Terebinthinæ in Burns and Scalds, for I am not sure it is from from its stimulus, there is a circumstance appears to me to have been overlooked by Dr. Kinglake as well as yourselves; indeed, the profession in general; and that is, the terebinthinate treatment of burns and scalds is not the discovery of Dr. Kentish. We are certainly obliged to him for a revival of the practice; how far his internal treatment is an improvement or original, I shall not attempt to investigate, as I wish to avoid controversy.

In the compilation of Heister, as Mr. Pott used to term it, oleum terebinthinæ is thus spoken of, in the curation of burns and scalds, "Oleum terebinthinæ has very good effects in this case, if you apply it in time, and repeat it frequently;" and there is no doubt of its being applied hot, as well as the oxycrate and salt there mentioned.— Oxycratum cum sale decoctum calidumque." The heating external treatment of burns then is no innovation, as has been generally and terrifically understood, and heat in any form recommended by Dr. Kentish do I see omitted in Heister; for in what he (Heister) calls the emollient practice, the cataplasms are ordered to be "frequently laid on upon the affected part, as warm as they can be endured." *

As to the heating of plaisters, as suggested by Mr. Harrold, Medical Journal, vol. xv. p. 148, though not mentioned by Heister, yet unguents spread on linen cloth or tow, and held contra ignem before their application, he will find spoken of by other authors.

Dr. Kentish attributes the good effects of ol. tereb. to its stimulus, Heister to its astringency; I do not pretend to say which is correct. The theory of Heister, I am apprehensive, will have but few advocates, as it lacks the plausibility of Dr. Kentish's, yet the stimulant doctrine is, I conceive, liable to objection. My apprentice bathed the back part of his hand ten minutes with hot ol. tereb.; he found little or no sensation, (saving cold) which must have been the case, if the heated ol. tereb. approximated in degree to the stimulus of fire, so much so, as only to be a shade or two less, as described by Dr. Kentish; besides, the disengaged caloric produces vesications, and heated ol. tereb. as fire in a lesser degree, must still go on with the vesication, though in a lesser degree, and yet it is said to prevent vesication entirely. Other objections, I conceive, would

* Sydenham was in favor of fomentations in burns and scalds as an emollient, with water as hot as the patient can bear, till the pain and heat entirely disappear.

would hold good as to the stimulant theory. But this I leave to others; my motive for troubling you with this, is to request that practitioners would pay a little more attention to those authors who are out of fashion and laid upon the shelf, and not suffer themselves to be coaxed by an old practice in a modern garb.

The latitude of ol. tereb. as an external application in burns and scalds, is well exemplified in Heister, and I should recommend his astringent and emollient practice, as well as *internal* treatment, to the consideration of Dr. Kinglake.

I am, &c.,

W. MARSON.

